# Multiscale Analysis of Mechanical Properties of 3D Orthogonal Woven Composites with Randomly Distributed Voids

**DOI:** 10.3390/ma14185247

**Published:** 2021-09-13

**Authors:** Yaohua Gong, Tao Huang, Xun’an Zhang, Yongyong Suo, Purong Jia, Shuyi Zhao

**Affiliations:** 1 School of Mechanics, Civil Engineering and Architecture, Northwestern Polytechnical University, Xi’an 710129, China; gongyh@mail.nwpu.edu.cn (Y.G.); jiaoping@nwpu.edu.cn (X.Z.); yysuo09@mail.nwpu.edu.cn (Y.S.); prjia@nwpu.edu.cn (P.J.); 2 Department of System Engineering and Integration, AECC Commercial Aircraft Engine Co., LTD., Shanghai 201100, China; zsy_gtlx@163.com

**Keywords:** 3D woven composites, multiscale analysis, voids, mechanical properties

## Abstract

Voids are common defects in 3D woven composites because of the complicated manufacturing processes of the composites. In this study, a micro–meso multiscale analysis was conducted to evaluate the influence of voids on the mechanical properties of three-dimensional orthogonal woven composites. Statistical analysis was implemented to calculate the outputs of models under the different scales. A method is proposed to generate the reasonable mechanical properties of the microscale models considering randomly distributed voids and fiber filaments. The distributions of the generated properties agree well with the calculated results. These properties were utilized as inputs for the mesoscale models, in which void defects were also considered. The effects of these defects were calculated and investigated. The results indicate that tensile and shear strengths were more sensitive to the microscale voids, while the compressive strength was more influenced by mesoscale voids. The results of this study can provide a design basis for evaluating the quality of 3D woven composites with void defects.

## 1. Introduction

The past few decades have witnessed significant advances in the application of 3D woven composites owing to their superior mechanical properties, such as high specific stiffness/strength, high damage tolerance and high energy absorption capability [1,2,3]. The wide and extended use of these composites requires higher mechanical property standards. However, defects such as voids in the matrix are almost inevitable during the molding process because of the complex spatial structure of the woven composite. The presence of random void defects can significantly reduce the mechanical properties and cause the fluctuation of mechanical properties [4,5,6]. Moreover, the randomly distributed fiber filaments in microscale models also significantly influence the mechanical performance of composites [7]. Thus, this paper presents a micro–mesoscale analysis to evaluate the influence of randomly distributed voids on the mechanical behaviors of three-dimensional orthogonal woven composites (3DOWCs), considering randomly distributed fiber filaments in bundles.

Three-dimensional woven composites have complicated spatial structures, and their heterogeneity mainly reflects in micro and mesoscales. In the microscale model, the fiber bundles, also treated as unidirectional (UD) composites, of 3D woven composites consist of a matrix and thousands of randomly distributed fiber filaments [7,8,9]. In the mesoscale model, the weave architecture, orientations of different fiber bundles and the matrix are incorporated [10,11,12,13,14]. In both scales, the representative volume element (RVE) models are treated as periodic structures and utilized to calculate the mechanical properties by applying periodic boundary conditions (PBCs). Outputs from microscale models are used as inputs for mesoscale models. For example, Wang et al. [15] used a hexagonal-array microscale model to predict effective elastic properties of fiber bundles. The outcomes were used as inputs for predicting the moduli of a 3D orthogonal woven fiber-reinforced polymer matrix composite. Zhou et al. [16] conducted a progressive damage analysis for 2D plain woven composites. The authors utilized RVE models of hexagonally arranged fiber filaments in microscale analysis, calculated the elastic properties and strength properties, and then applied the results to mesoscale models. In recent years, an increasing number of researchers have used randomly distributed fiber models instead of regularly arranged models in microscale analysis. Representative volume elements with more fibers can capture the variation in strength, especially in transverse directions. Vajari [17,18] used randomly distributed models to study the influence of micro-voids in the matrix on biaxial strength properties and failure modes. Wang [19] studied the transverse tensile strengths of UD composites, considering thermal residual stress, using randomly distributed fiber models, and found variations due to the distributions. The arrangement of fibers is uncertain in advance. Randomly distributed fibers in micro-scale models will result in different elastic and strength properties. Thus, Monte-Carlo simulations are implemented in this paper to study the influence of randomness on the mechanical behaviors. To obtain a stable result, a process should be repeated numerously because of uncertainties [20,21,22]. In addition to the randomly distributed fibers, there are many other uncertainty factors, such as material properties, volume fractions, random cross sections and randomly distributed voids. Tao et al. [7,23] proposed a multiscale simulation to quantify the effects of numerous uncertainties on mechanical properties for 3DOWCs, including fiber and void distributions, modulus of the matrix, fiber volume contents and fiber bundle dimensions. Zhou et al. [24] coupled the multiscale method and perturbation to study the variability in the effective elastic properties of composites with random material properties. Shi et al. [14] used Weibull distribution material properties to conduct a multiscale damage analysis for 2.5D fiber-reinforced ceramic matrix composites. The results showed significant relationships existed between the constituent properties and the effective mechanical properties. Roham and Mohammad [25] conducted a progressive damage analysis for composite vessels, considering several uncertainties, including fiber volume fraction, winding angle and material strength properties. The importance of considering uncertainties is emphasized using statistical analysis. Guo et al. [26] proposed a random weft cross-section model based on experimental observations. The predicted results agreed well with the experimental results.

Void defect is also a common uncertainty generated from manufacturing processes, and it can mainly be classified into two types: microscale and mesoscale void defects. Recently, several researchers have focused on the effects of these defects on the mechanical performance of composites. For microscale defects, Dong [27] provided a practical method for predicting the effects of process-induced voids on the mechanical properties of carbon fiber/epoxy composites using the RVE model. Tensile and interlaminar shear strengths were calculated and compared with available experimental data. Jiang et al. [28] compared the axial stiffness and strength properties of a single-fiber bundle in UD composites with and without void defect based on a three-representative unit cell model. Carrera [29] used a 1D finite element model based on the Carrera unified formulation to evaluate the influence of voids on UD composites. Hyde et al. [30] conducted comprehensive research on the effects of micro-voids on the strength of UD composites. The effects of void shapes, void volume fractions and void orientations were considered. For mesoscale defects, Dong and Huo [31] studied the elastic properties of 3D braided composites with internal defects using a two-scale finite element model. Huang and Gong [32] used a similar method to predict the void effects on the effective elastic properties of 3DOWC. Gao et al. [13] recently predicted the mechanical properties of 3D braided composites with void defects, establishing a constitutive model and a finite element model of 3D braided composites. The results provided the design basis for evaluating the influence of void defects on mechanical behaviors.

An integral part of the multiscale finite element research is to build a microscale RVE that considers uncertainties. Monte-Carlo simulations are then conducted to obtain the statistical distributions of required mechanical properties. Some researchers used mean values as inputs for higher-scale analysis, while other researchers considered the distributions of these parameters and then generated distributed inputs for higher-scale analysis. However, most researchers ignored the relationships between these mechanical properties; the relationships are not stated in their papers. Thus, the main goal of this paper is to devise a multiscale approach to evaluate the influence of microscale and mesoscale void defects on the mechanical properties of 3DOWCs. The relationships between microscale properties are considered. The rest of this paper is organized as follows: Section 2 describes the multiscale models and boundary conditions. Section 3 introduces the constitutive models and failure criteria used in this paper. Section 4 provides the results and discussion on the multiscale analysis. Section 5 presents the concluding remarks and future work.

## 2. Multiscale Models and Methods

**Figure 1:**
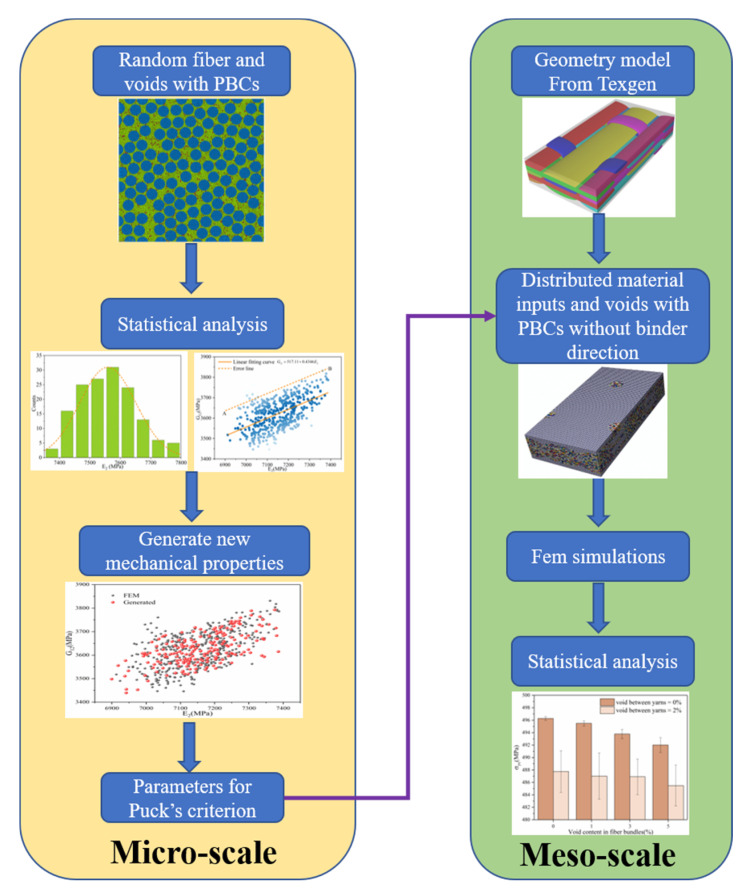
Multiscale stochastic modeling framework.

A 3D orthogonal woven fiber-reinforced polymer matrix composite was investigated in this study, and the material properties of the basic components are listed in Table 1. The 3DOWC had a one-by-one weave architecture. In the thickness direction, there were two layers in the warp direction and three layers in the weft direction. Figure 1 illustrates the framework of the multiscale stochastic model used in this study. Multiscale models with randomly distributed fiber filaments and voids were generated. Statistical analysis was conducted to obtain the correlations between the mechanical properties, and the results are described in Section 4. Then, based on the above correlations, new mechanical properties were generated as the input parameters for mesoscale models. Finally, statistical analysis was conducted to evaluate the effect of voids on the mechanical properties of 3DOWCs.

### 2.1. Microscale Model for UD Composites

**Figure 2:**
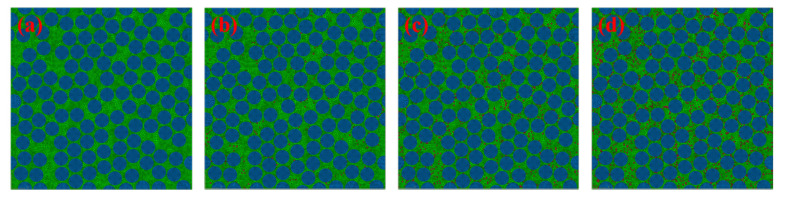
Microscale finite element model of randomly distributed fibers with void contents: (**a**) 0%, (**b**) 1%, (**c**) 3% and (**d**) 5%.

In the microscale model, randomly distributed fibers were considered. Since the focus of this study is to evaluate the influence of void defects on mechanical properties, randomly distributed voids were also implemented in the matrix. Therefore, the material properties of fibers and matrix remained constant, and the randomly distributed fibers and voids were the variables. CATIA V5R21 (Vélizy-Villacoublay, France) was used to generate the randomly distributed geometry model of UD composites considering the periodicity, and then HyperMesh 14.0(Altair Engineering, Inc., Troy, MI, USA) was used for meshing. Corresponding nodes were on opposite surfaces to ensure the following application of PBCs. There are two ways to generate the void defects: One is considering the real geometry of voids and distributions and then establishing the finite element model; the other one is randomly selecting the matrix elements, putting them into one set and modifying the material properties of these elements. These two methods result in different void densities or numbers of voids, depending on the grid density. According to Vajari [17,18], increasing the number of matrix voids or void density in the model has no significant effect on the strengths or moduli but alters the crack path. In this study, the latter method was adopted, and its feasibility has been verified by many researchers [28,29,31,32]. To generate the random voids in the matrix, a Python (Python Software Foundation, Beaverton, OR, USA) script was used to modify the input file for ABAQUS 2020(Dassault Systemes Simulia Corp., Johnstone, RI, USA). Random elements were selected from the matrix set to the void set until the content of voids reached the set value. This way, 150 models were generated. Illustrations are shown in Figure 2. The fiber diameter was 7 μm, and the RVE model was 90 μm in length and width and 2 μm in thickness. Over one hundred fibers filaments are included in microscale model. Fibers, voids and matrix were meshed with eight-node linear hexahedral reduced integration elements (C3D8R). There were 84,519 elements and 113,888 nodes in this model. After the mesh sensitivity analysis, accurate mechanical properties could be obtained using the mesh size.

### 2.2. Mesoscale Model for 3DOWCs

**Figure 3:**
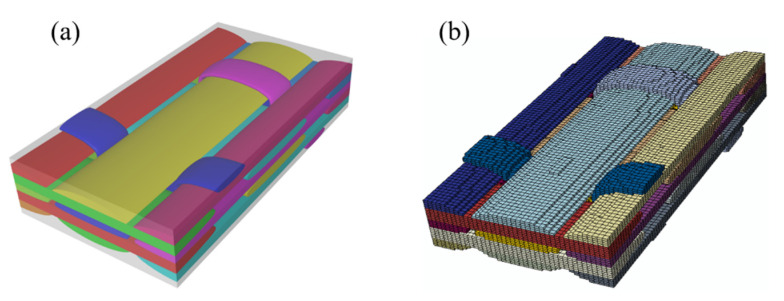
Textile geometry (**a**) and voxel mesh (**b**) of the three-dimensional orthogonal woven composites.

The 3DOWC has a relatively complicated weave architecture, which may cause difficulty in the periodic meshing process. In this study, the textile geometry models of 3DOWC RVEs were generated using TexGen software (version 3.10.0, University of Nottingham, Nottingham, UK) [33]. Compared with the consistent meshing method that considers the real geometry of the fiber bundle section, the voxel meshing method has advantages in periodic meshing with a balance of efficiency and accuracy. Liu [34] studied the strength properties of 3DOWCs based on the voxel model, and the results agreed well with the experimental results. Owing to the efficiency and accuracy of this method, the voxel meshing method for the textile geometry model generated from TexGen was utilized in this study. Figure 3a,b present the mesoscale geometry model and voxel meshing for fiber bundles, respectively. The generation technique of voids in mesoscale model of 3DOWCs is the same with that in microscale model as is discussed in Section 2.1. The material orientations for each element of fiber bundles were automatically assigned by TexGen. The numbers of mesh seeds were 40, 80 and 32 in the warp, weft and thickness directions, respectively. Eight-node linear hexahedral reduced integration elements were assigned for fiber bundle and matrix components. A total of 102,400 elements and 109,593 nodes were included in this model.

### 2.3. Periodic Boundary Conditions

To satisfy the boundary displacement and boundary stress continuity of the RVE, PBCs should be employed to obtain accurate mechanical behavior. The PBCs should satisfy the following equations:

(1) $$\left\{ \begin{array}{l} u_{i}(a_{1},x_{2},x_{3})-u_{i}(-a_{1},x_{2},x_{3})=2a_{1}\varepsilon_{i1}^{0}\begin{array}{c} -a_{2}\leq x_{2}\leq a_{2}\\ -a_{3}\leq x_{3}\leq a_{3} \end{array}\;\\ u_{i}(x_{1},a_{2},x_{3})-u_{i}(x_{1},-a_{2},x_{3})=2a_{2}\varepsilon_{i2}^{0}\begin{array}{c} -a_{1}\leq x_{1}\leq a_{1}\\ -a_{3}\leq x_{3}\leq a_{3} \end{array}\\ u_{i}(x_{1},x_{2},a_{3})-u_{i}(x_{1},x_{2},-a_{3})=2a_{3}\varepsilon_{i3}^{0}\begin{array}{c} -a_{1}\leq x_{1}\leq a_{1}\\ -a_{2}\leq x_{2}\leq a_{2} \end{array} \end{array}\right.$$

Here, $a_{1}$, $a_{2}$ and $a_{3}$ represent the RVE length in the first, second and third directions, respectively; $\varepsilon_{i1}^{0}$ denotes the strain in the i direction applied on the RVE surface in the first direction; and $u_{i}$ denotes the displacement in the i direction.

**Figure 4:**
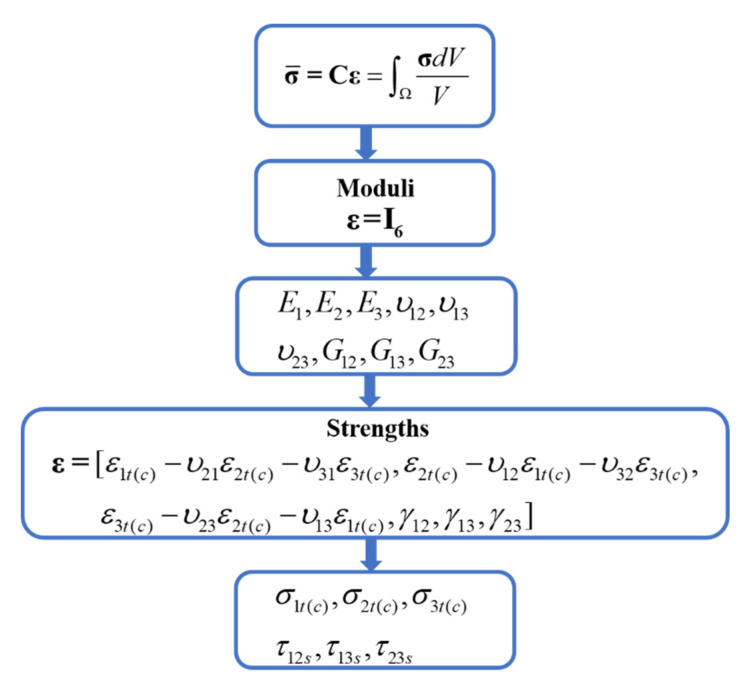
Program of applying periodic boundary conditions for determining elastic properties and strength properties.

The PBCs described in this paper were applied using nodes constraint equations by Python script in ABAQUS. To obtain the RVE mechanical properties, including moduli and strengths, separate PBCs should be considered. Figure 4 presents the program of applying the PBCs for the microscale model. In the first step, the effective moduli of the RVE are calculated in the post-processing stage using $\bar{\sigma }=\int_{\Omega }\frac{\sigma dV}{V}$. Here, $I_{6}$ is the unit matrix. Details for computing tensor C and the related elastic moduli can refer to Barbero [35]. Then, PBCs for axial and biaxial strength analysis are applied with the Poisson effect. The normal and shear stresses of the RVE are computed from the resultant normal and tangential forces acting on the surfaces divided by the cross section at every sub-step in ABAQUS. Finally, the strength properties are extracted from σ−ε curves in the direction of interest. For the microscale RVE model, PBCs in all directions are applied, while for mesoscale analysis, PBCs in the thickness direction are ignored because of the full-thickness RVE model [36]. One corner for each RVE model is fixed in all directions to remove the rigid motion.

### 2.4. Distance Function for Generating Mechanical Properties of Microscale Model

The randomness of filament and void distributions will result in different mechanical properties for microscale models. In order to evaluate these parameters under certain void content, fitting functions are implemented to show the distributions of these mechanical properties. However, some of these parameters are positive correlated or negative correlated, see Section 4.1. Thus, it is inappropriate to directly use the fitting functions to generate the parameters as inputs for higher scale models. 

**Figure 5:**
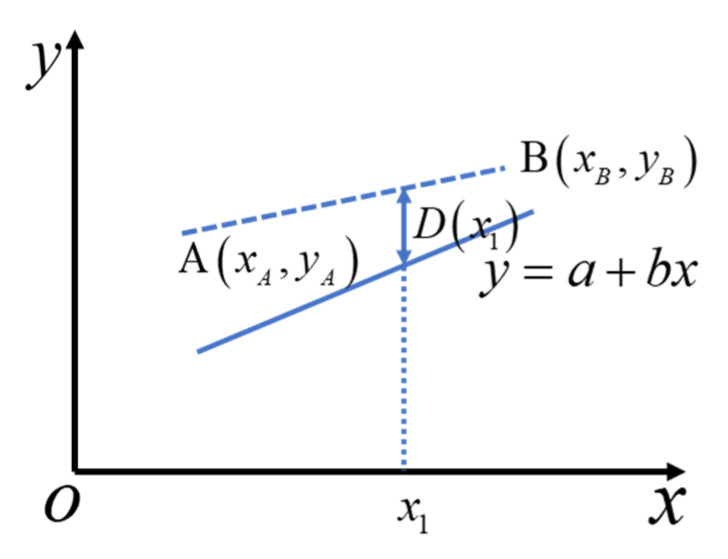
Diagram for determining the relationship between x and y.

To tackle this problem, a distance function is proposed here. After sufficient simulations, all elastic and strength properties are obtained. Thus, the scattered diagrams can be drawn between every two above parameters. As shown in Figure 5, we assume the fitting line between the two parameters is y=a+bx. Next, two points A and B which forms the error line should be found manually. It should be ensured that nearly all the scatter points are below the error line. Define the distance between the two lines at arbitrary point $x_{1}$ as $D\left(x_{1}\right)$. Then the relationship between x and y can be written as follows:

(2) y=a+bx+rand(−D(x),D(x)),

where *x* and *y* represent the mechanical parameters, *a* and *b* are the linear fitting parameters of the fitting line, and the random function rand(c,d) returns a random value between c and d. D(x) is the distance function, which returns the linear distance value in y-direction between the linear fitting line and the error lines.

## 3. Failure Criteria and Constitutive Models

### 3.1. Failure Initiation Criteria

For the microscale model, the fiber component is regarded as a transversely isotropic material. In actual situations, no damage behavior is observed in transverse directions; thus, the maximum stress failure criteria are used to describe the failure behavior of fiber filaments because of their brittle damage behavior, as shown in Equation (3):

(3) $$\begin{array}{l} f_{f1t}=\sigma_{11}/X_{ft}\geq 1\\ f_{f1c}=\left|\sigma_{11}\right|/X_{fc}\geq 1^{\prime } \end{array}$$

where $X_{t}$ and $X_{c}$ are failure stresses for fiber tensile and compressive failure modes, respectively, and $\sigma_{11}$ denotes the stress of the fiber component in the fiber longitudinal direction.

Matrix materials in fiber bundles (microscale) and between fiber bundles (mesoscale) were modeled as an isotropic material, and the modified Drucker–Prager yield model developed by Lubliner et al. [37] and Lee and Fenves [38] was applied to estimate the failure as follows:

(4) $$f_{m}=\frac{1}{1-\alpha }\left(\sqrt{3J_{2}}+\alpha I_{1}+\beta \langle \sigma_{1}\rangle \right)-\sigma_{xc}\geq 0,$$

where $I_{1}$ is the first invariant of the stress tensor; $J_{2}$ is the second invariant of the deviatoric stress tensor; $\sigma_{1}$ is the maximum principal stress; $\langle x\rangle =\frac{x+\left|x\right|}{2}$; α is the pressure-sensitivity parameter and the value 0.13 is adopted in is paper; and β is a function of tensile ($\sigma_{xt}$) and compressive ($\sigma_{xc}$) yield stress and is defined as follows:

(5) $$\beta =\frac{\sigma_{xc}}{\sigma_{xt}}\left(1-\alpha \right)-\left(1+\alpha \right)$$

The sudden stiffness degradation law is used to describe the damage behavior after failure initiation for the fiber filament and matrix components.

For the mesoscale model, the fiber bundles can be regarded as fiber-reinforced UD composites. Thus far, numerous failure criteria have been proposed to describe the failure initiation and damage evolution behavior for UD composites. Puck’s criterion, developed by Puck and Schürmann [39], ranks high among the several failure criteria in “World Wide Failure Exercise I and II” and was adopted in this study. Moreover, Gu and Chen [40] extended Puck’s inter-fiber failure (IFF) criterion, and the extended criterion was also considered in this study. Puck’s theory can be classified into two sets of equations: fiber failure (FF) and IFF.

The refined FF criteria were selected to identify the failure initiation for fiber damage under tensile and compressive loads as follows [41]:

(6) $$f_{t(c)}=\left|\frac{\sigma_{11}}{X_{t(c)}}-(\frac{\upsilon_{12}}{X_{t(c)}}-\upsilon_{12f}\frac{E_{1}}{E_{1f}}m_{\sigma f})(\sigma_{22}+\sigma_{33})\right|\geq 1$$

where $X_{t}$ and $X_{c}$ are the longitudinal tensile and compressive strengths of UD composites, respectively; $E_{1}$ and $E_{1f}$ are the longitudinal moduli of UD composites and fiber, respectively; $\upsilon_{12}$ and $\upsilon_{12f}$ are the Poisson ratios of UD composites and fiber, respectively; and $m_{\sigma f}$ is a magnification factor for the matrix stress caused by the mismatch in the moduli of matrix and fibers; in this study, $m_{\sigma f}=1.1$ was adopted, as suggested in [42,43].

**Figure 6:**
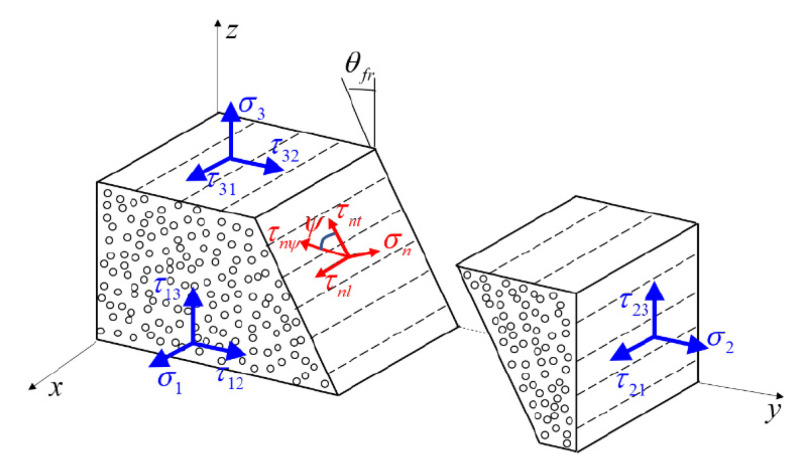
Stress components acting on potential fracture plane. (Blue stresses are in global coordinate system and red stresses are in local coordinate system.)

Puck’s IFF criterion is based on the Mohr–Coulomb theory, and failure will occur on the fracture plane where only shear stresses $\tau_{nt}$ and $\tau_{nl}$ and normal stress $\sigma_{n}$ exist, as shown in Figure 6. The IFF criterion is classified into two modes: matrix tension ($\sigma_{n}(\theta )\geq 0$) and matrix compression ($\sigma_{n}(\theta )<0$), which can be introduced as follows:

(7) $$f_{IFF}=\{\begin{array}{ll} \sqrt{{\left(\frac{1}{R_{⊥}^{A+}}-\frac{p_{⊥\psi }^{\left(+\right)}}{R_{⊥\psi }^{A}}\right)}^{2}\sigma_{n}^{2}(\theta )+{\left(\frac{\tau_{nt}(\theta )}{R_{⊥⊥}^{A}}\right)}^{2}+{\left(\frac{\tau_{nl}(\theta )}{R_{⊥∥}}\right)}^{2}}+\frac{p_{⊥\psi }^{\left(+\right)}}{R_{⊥\psi }^{A}}\sigma_{n}(\theta ) & \mathrm{for}\;\sigma_{n}(\theta )\geq 0\\ \sqrt{{\left(\frac{p_{⊥\psi }^{\left(-\right)}}{R_{⊥\psi }^{A}}\sigma_{n}(\theta )\right)}^{2}+{\left(\frac{\tau_{nt}(\theta )}{R_{⊥⊥}^{A}}\right)}^{2}+{\left(\frac{\tau_{nl}(\theta )}{R_{⊥∥}}\right)}^{2}}+\frac{p_{⊥\psi }^{\left(-\right)}}{R_{⊥\psi }^{A}}\sigma_{n}(\theta ) & \mathrm{for}\;\sigma_{n}(\theta )<0 \end{array}$$

With:

(8) $$\begin{array}{l} \frac{p_{⊥\psi }^{\left(+,-\right)}}{R_{⊥\psi }^{A}}=\frac{p_{⊥⊥}^{\left(+,-\right)}}{R_{⊥⊥}^{A}}\cos^{2}\varphi +\frac{p_{⊥∥}^{\left(+,-\right)}}{R_{⊥∥}^{A}}\sin^{2}\varphi ’\cos^{2}\varphi =\frac{\tau_{nt}^{2}(\theta )}{\tau_{nt}^{2}(\theta )+\tau_{nl}^{2}(\theta )}\\ R_{⊥⊥}^{A}=\frac{Y_{c}}{2(1+p_{⊥⊥}^{-})},R_{⊥∥}^{A}=S_{21} \end{array}$$

(9) $$\begin{array}{l} \sigma_{n}(\theta )=\sigma_{2}\cos^{2}\theta +\sigma_{3}\sin^{2}\theta +2\tau_{23}\sin\theta \cos\theta \\ \tau_{nt}(\theta )=(\sigma_{3}-\sigma_{2})\sin\theta \cos\theta +\tau_{23}(\cos^{2}\theta -\sin^{2}\theta )\\ \tau_{nl}(\theta )=\tau_{12}\cos\theta +\tau_{13}\sin\theta \end{array}$$

The seven parameters $R_{⊥⊥}^{A},R_{⊥∥}^{A},R_{⊥}^{A+},p_{⊥∥}^{\left(+\right)},p_{⊥∥}^{\left(-\right)},p_{⊥⊥}^{\left(+\right)},p_{⊥⊥}^{\left(-\right)}$ need to be determined before Puck’s criterion is implemented. According to Gu and Chen [40], UD composites are divided into three categories, resulting in different formulas to obtain the above parameters. Details can be found in ref. [40].

In Equation (7), $f_{IFF}$ is a function of the potential fracture angle θ, namely the fracture plane. The plane with the highest $f_{IFF}$ is the actual fracture plane. Thus, for an arbitrary stress state and uncertain material properties, the potential fracture angle should be determined before the failure onset is predicted. However, it is extremely difficult to obtain the analytical solution of fracture angle for such a 3D problem. Thus, the semi-analytical procedure is utilized. In this study, an extension and combination algorithm of the selective range golden section search (SRGSS) [44] and the semi-analytical algorithm (SAA) [45] was developed to accurately determine the fracture angle. The algorithm implemented in this paper is more accurate than the SRGSS and SAA. However, a detailed description of the algorithm is beyond the scope of this paper and is thus not presented here. Once the fracture angle is obtained, the global maximum value $f_{IFF}$ is calculated, and the matrix damage is initiated when $f_{IFF}$ reaches 1.

Once the fiber damage and matrix damage starts, damage evolution processes begin and the equivalent stresses and strains of the fiber and matrix are defined as follows [46]:

(10) $$\begin{array}{l} \sigma_{eq}^{f}=\sqrt{\sigma_{11}^{2}+\sigma_{22}^{2}+\sigma_{33}^{2}},\;\sigma_{eq}^{m}=\sqrt{{\langle \sigma_{n}\rangle }^{2}+\tau_{nt}^{2}+\tau_{nl}^{2}}\\ \varepsilon_{eq}^{f}=\sqrt{\varepsilon_{11}^{2}+\varepsilon_{22}^{2}+\varepsilon_{33}^{2}},\;\varepsilon_{eq}^{m}=\sqrt{{\langle \varepsilon_{n}\rangle }^{2}+\varepsilon_{nt}^{2}+\varepsilon_{nl}^{2}} \end{array}$$

With:

(11) $$\begin{array}{l} \varepsilon_{n}=\varepsilon_{2}\cos^{2}\theta +\varepsilon_{3}\sin^{2}\theta +2\varepsilon_{23}\sin\theta \cos\theta \\ \varepsilon_{nt}=(\varepsilon_{3}-\varepsilon_{2})\sin\theta \cos\theta +\varepsilon_{23}(\cos^{2}\theta -\sin^{2}\theta )\\ \varepsilon_{nl}=\varepsilon_{13}\sin\theta +\varepsilon_{13}\cos\theta \end{array}$$

With $\varepsilon_{eq}^{0}$ and $\varepsilon_{eq}^{f}$ as the equivalent strains of damage initiation and complete damage, respectively, the equivalent strain $\varepsilon_{eq}^{0}$ for FF and IFF can be written as:

(12) $$\varepsilon_{eq}^{f0}=\frac{\varepsilon_{eq}^{f}}{f_{t(c)}},$$

(13) $$\varepsilon_{eq}^{m0}=\frac{\varepsilon_{eq}^{m}}{f_{IFF}}.$$

As for the equivalent strain of complete damage for IFF, the energy-based damage law under mixed-mode is built with the element characteristic length to alleviate mesh dependency during material softening:

(14) $${\left(\frac{g_{n}L}{G_{mt(c)}}\right)}^{\xi }+{\left(\frac{g_{nt}L}{G_{23c}}\right)}^{\xi }+{\left(\frac{g_{nl}L}{G_{12c}}\right)}^{\xi }=1’$$

where $g_{n},g_{nt}$ and $g_{nl}$ are the strain energy density of the corresponding stress component, respectively; $G_{mt(c)}$ is the energy dissipated during damage for transverse tensile and compressive directions, respectively; $G_{23c}$ and $G_{12c}$ are in-plane and out-of-plane dissipated energies for the corresponding directions; L is the characteristic length; ξ is a material parameter, set as 2 [46] in this paper.

The strain energy density associated with each effective stress component due to complete damage is defined as:

(15) $$g_{j}^{f}=\int_{0}^{\varepsilon_{j}^{f}}\sigma_{j}d\varepsilon_{j}\approx \frac{1}{2}\sigma_{j}^{0}\varepsilon_{j}^{f}=\frac{1}{2}\sigma_{j}^{0}\beta \varepsilon_{eq}^{mf},\;j=n,nt,nl,$$

where $\sigma_{j}^{0}$ is the stress component on the fracture plane when the damage is initiated, and $\beta_{j}$ represents the mix-mode ratio and can be expressed as:

(16) $$\beta_{n}=\frac{\langle \varepsilon_{n}\rangle }{\varepsilon_{eq}^{m}},\;\beta_{nt}=\frac{\varepsilon_{nt}}{\varepsilon_{eq}^{m}},\;\beta_{nl}=\frac{\varepsilon_{nl}}{\varepsilon_{eq}^{m}}.$$

Thus, the equivalent failure strain $\varepsilon_{eq}^{mf}$ can be formulated by substituting Equations (15) and (16) into Equation (14):

(17) $$\varepsilon_{eq}^{mf}=\frac{2}{L}{\left[{\left(\frac{\sigma_{n}^{0}\beta_{n}}{G_{mt(c)}}\right)}^{\xi }+{\left(\frac{\sigma_{nt}^{0}\beta_{nt}}{G_{23c}}\right)}^{\xi }+{\left(\frac{\sigma_{nl}^{0}\beta_{nl}}{G_{12c}}\right)}^{\xi }\right]}^{-1/\xi }$$

The IFF damage variable is given by the following expression based on the bilinear model:

(18) $$d_{m}=\frac{\varepsilon_{eq}^{mf}\left(\varepsilon_{eq}^{m}-\varepsilon_{eq}^{m0}\right)}{\varepsilon_{eq}^{m}\left(\varepsilon_{eq}^{mf}-\varepsilon_{eq}^{m0}\right)}$$

Similarly, the equivalent strain of complete damage for FF can be obtained through the same procedure.

In the damage evolution process, the generalization of the Duvaut–Lions regularization model [47] is adopted for the mesoscale model to improve the convergence of the numerical calculation and smoothen the stiffness degradation. Thus, the viscous damage variable is defined as follows:

(19) $$\overset{\cdot }{d_{i}^{v}}=\frac{1}{\eta }(d_{i}-d_{i}^{v})$$

### 3.2. Damage Constitutive Model

In this paper, the damage modes for fiber bundles are mainly classified into four categories: longitudinal tensile damage $D_{ft}$, longitudinal compressive damage $D_{fc}$, transverse tensile damage $D_{mt}$ and transverse compressive damage $D_{mc}$. When the material is damaged, its stiffness is degraded. If the damage occurs only in the y direction ($\theta_{fr}=0$), the material can still bear load in the z direction. The degraded stiffness matrix can therefore be expressed as follows:

(20) $$C_{D}=\left[\begin{array}{cccccc} C_{11}(1-D_{f}) & C_{12}(1-D_{f})A_{1}A_{4} & C_{13}(1-D_{f})A_{2}A_{3} & & & \\ & C_{22}A_{1}A_{2} & C_{23}(1-D_{mt})(1-D_{mc}) & & 0 & \\ & & C_{33}A_{3}A_{4} & & & \\ & & & C_{44}(1-D_{f})A_{1}A_{2} & & \\ & SYM & & & C_{55}(1-D_{f})A_{3}A_{4} & \\ & & & & & C_{66}(1-D_{mt})(1-D_{mc}) \end{array}\right],$$

where, $D_{f}=1-\left(1-D_{ft}\right)\left(1-D_{fc}\right)$, $A_{1}=1-D_{mt}\cos\theta_{fr}$, $A_{2}=1-D_{mc}\cos\theta_{fr}$, $A_{3}=1-D_{mt}\sin\theta_{fr}$ and $A_{4}=1-D_{mc}\sin\theta_{fr}$.

Thus, the stresses are calculated using the following equation:

(21) $$\sigma =C_{D}:\varepsilon$$

The Jacobian matrix can be formulated by differentiating Equation (21) as follows:

(22) $$\frac{\partial \Delta \sigma }{\partial \Delta \varepsilon }=C_{D}+\left(\sum_{i}\frac{\partial C_{D}}{\partial D_{i}}\frac{\partial D_{i}}{\partial \varepsilon_{eq}}\frac{\partial \varepsilon_{eq}}{\partial \varepsilon }\right):\varepsilon \left(i=ft,fc,mt,mc\right)$$

## 4. Results and Discussion

### 4.1. Microscale Model

**Figure 7:**
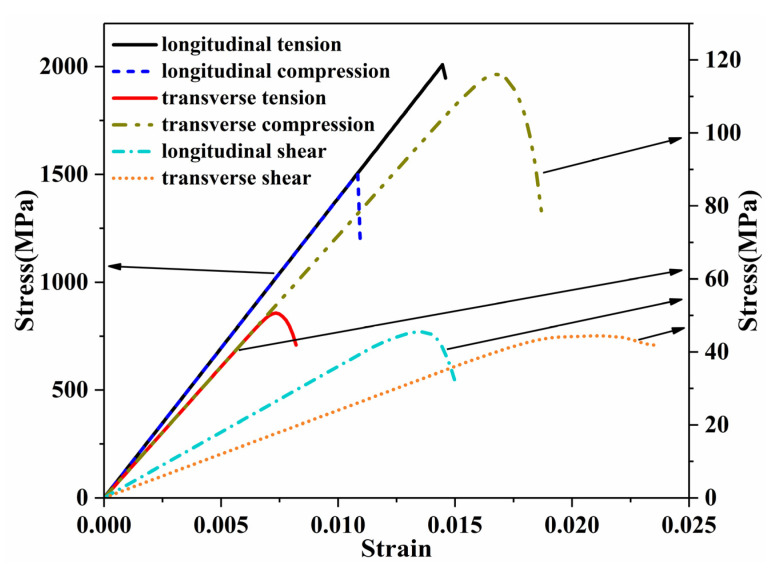
Typical stress–strain curves of unidirectional composite (3% void content) under different uniaxial loadings.

**Figure 8:**
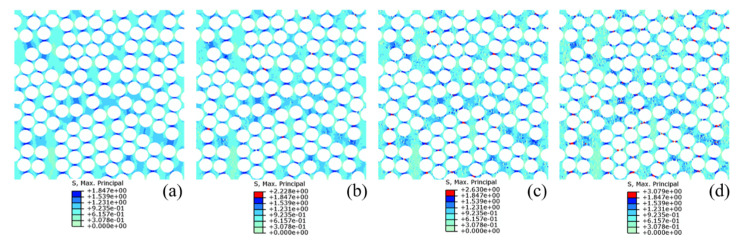
Maximum principal stress plots of microscale matrix component under transverse tensile load: (**a**) 0%, (**b**) 1%, (**c**) 3% and (**d**) 5%.

**Figure 9:**
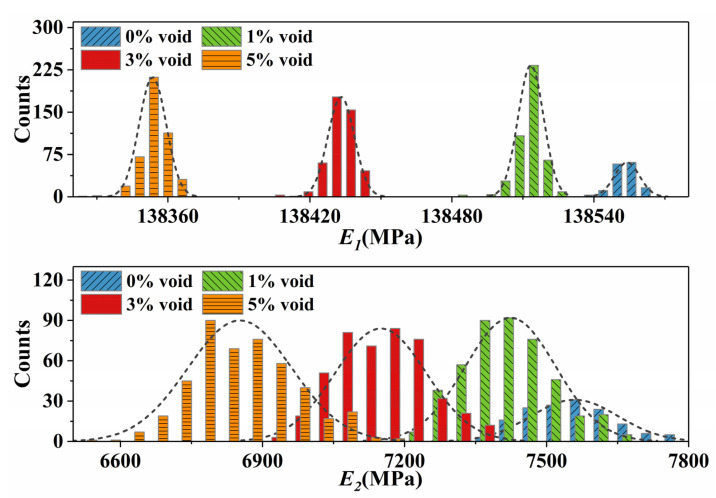
Counts distributions of *E*_1_ and *E*_2_ under different void contents.

Figure 7 displays the typical stress–strain curves of UD composites with 3% void content under different uniaxial loadings. The modulus, strength and energy release rate properties were all obtained from the curves. In this study, 150 randomly distributed microscale models were established. For each non-zero void content (1%, 3% and 5%), three models with randomly distributed voids were established for each microscale model. Therefore, there were a total of 150 random models without voids and 1350 random models with voids. Figure 8 gives the maximum principal stress plots for one microscale model with different void contents under transverse tensile loads. The fiber component is hidden in the figure. The presence of voids does not affect the general stress distribution but will cause discontinuities of the distribution. Meanwhile, a higher stress concentration occurs between two adjacent fiber filaments in the load direction, at which the matrix elements will be damaged compared with the void-free case. Figure 9 presents the counts distribution histograms of $E_{1}$ and $E_{2}$ of the UD composite with various void contents, and Gaussian distributions are implemented to fit the probability density functions, which will be utilized for generating mechanical properties in the mesoscale models. It should be noted when the Gaussian distribution is implemented in mesoscale model, the generated values should be in the minimum-maximum range, obtained from finite element simulations, in order to avoid the appearance of too large or too small values. The statistical parameters of Gaussian distributions for all mechanical properties are listed in Appendix A (Table A1).

**Figure 10:**
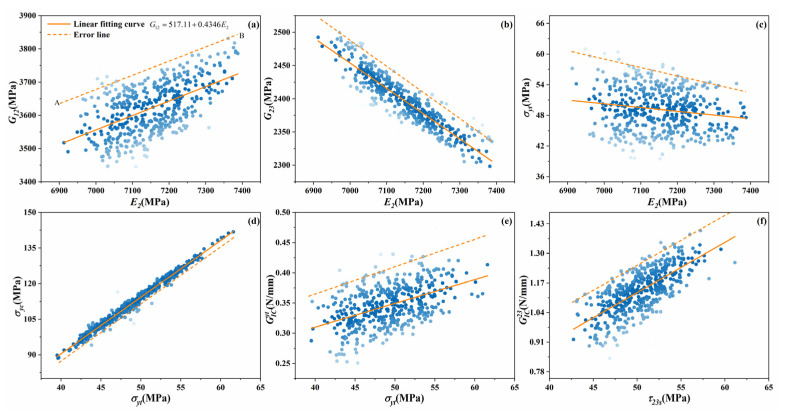
Relationships between the mechanical properties of microscale model with 3% void content.

Even though all the mechanical parameters follow the Gaussian distributions, they cannot be directly utilized as inputs for the mesoscale analysis because there are correlations among these parameters. For instance, Figure 10 displays the relations between $\left(G_{12},E_{2}\right)$, $\left(G_{23},E_{2}\right)$,$\left(\sigma_{yt},E_{2}\right)$,$\left(\sigma_{yc},\sigma_{yt}\right)$,$\left(G_{IC}^{yt},\sigma_{yt}\right)$ and $\left(G_{IC}^{23},\tau_{23s}\right)$ when the void content is 3%. In Figure 10a, the blue scattered bubbles denote the distributions of transverse modulus and longitudinal shear modulus. The solid line denotes the linear fitting curve, and the dashed error line is used to measure the deviation, which is also reflected in the bubble color intensity. The further away from the fitting curve, the lighter the bubble color. Upward and downward trends can be observed; therefore, the mechanical parameters cannot be directly and separately obtained from Gaussian distributions in the case of unreasonable situations such as UD composites with high transverse tensile strength and low transverse compressive strength.

Owing to the above reasons, Gaussian distributions were utilized for parts of the mechanical properties $E_{1},E_{2},\sigma_{xt},\sigma_{xc},G_{IC}^{xt}$ and $G_{IC}^{xc}$ as basic inputs for mesoscale analysis. Other mechanical properties were calculated based on the above parameters using Equation (2). 

**Figure 11:**
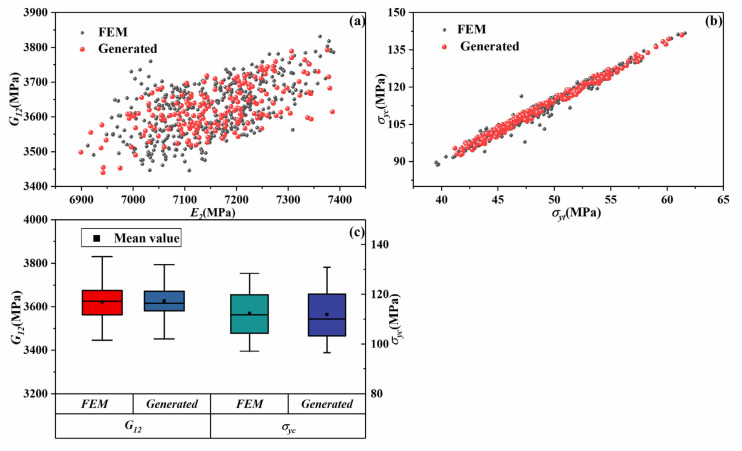
Distributions of calculated and generated mechanical properties. (**a**,**b**) represent the examples of calculated and generated results; (**c**) shows the distributions of two mechanical properties.

To predict the mechanical properties for unknown microscale models, Python scripts were utilized to generate all the microscale properties using the abovementioned method. Figure 11a,b displays the distributions of calculated results and generated results. Figure 11c gives the box plots of the distributions of mechanical properties. From the figures, the distributions of the generated mechanical properties agree well with the numerical results. Hence, the method proposed in this paper is accurate enough for predicting the mechanical properties considering randomly distributed fibers and void defects.

**Figure 12:**
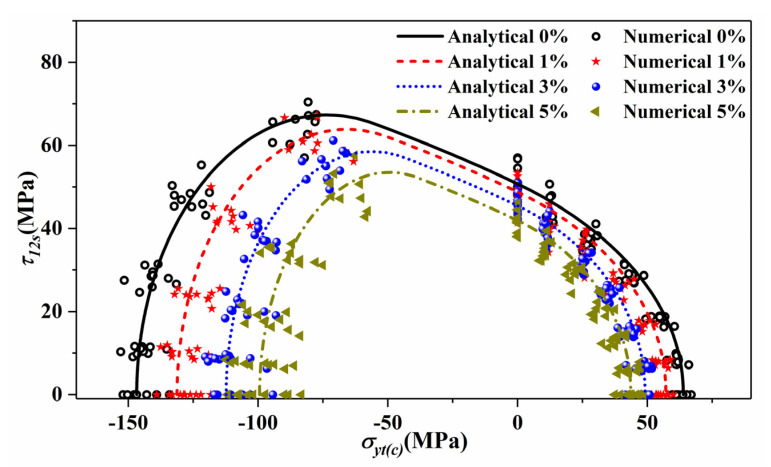
Failure envelopes of microscale model in $\sigma_{2}-\tau_{12}$ stress space under different void contents between calculated and theoretical results.

In the next scale analysis, the UD composites will be regarded as transverse isotropic materials, and Puck’s criterion will be adopted to predict the failure onset. Thus, biaxial loading simulations for UD composites were conducted to verify the applicability of Puck’s criterion for predicting the strength properties under uncertain models and complex loading conditions. Figure 12 displays the failure envelopes in $\sigma_{2}-\tau_{12}$ stress space under different void contents. In this case, 10 random models were selected for each void content case, and 12 stress ratios were considered. The numerical results agree well with the analytical results, verifying the applicability of Puck’s criterion.

### 4.2. Mesoscale Model

**Figure 13:**
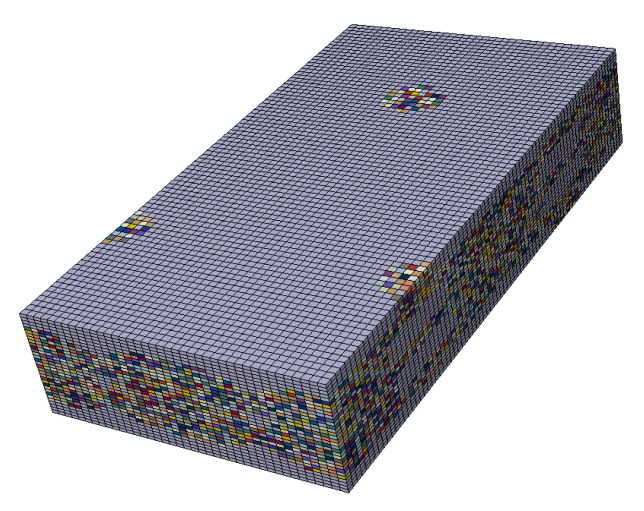
Representative volume element of mesoscale model with random material properties generated from microscale outputs.

The elastic properties and strength properties of 3DOWC mesoscale models in both warp and weft directions and in-plane shear properties were analyzed considering the randomly distributed voids in fiber bundles and between bundles. For the mesoscale models, the bundles were homogenized from the microscale model. Stochastic properties of bundles were generated based on the microscale models discussed above. Considering that the voids were randomly distributed in fiber bundles and that the fiber distribution is different at different places, the properties of fiber bundle elements were different from each other. In this study, we assumed that the distributions of voids and fibers differed among the bundle elements, which results in different mechanical properties. Thus, each bundle element had its own sections, and each section had unique mechanical properties generated from the constructed microscale models in the given void content case. Figure 13 shows the random material RVE model. Different color represents different materials or sections. A total of 50 groups of models were established for each load case to characterize the stochasticity of the mechanical properties. The above process was conducted using Python script by modifying the input files generated from ABAQUS.

**Figure 14:**
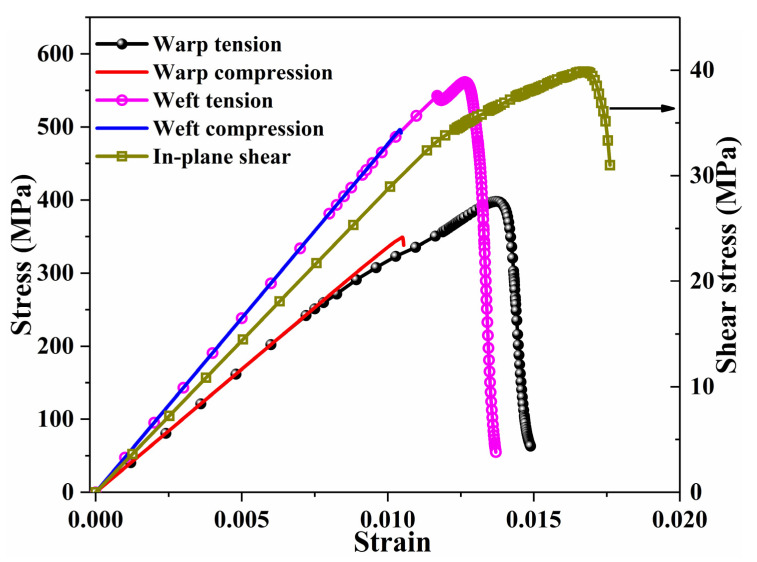
Typical stress–strain curves of three-dimensional orthogonal woven composite with no voids under different uniaxial loadings.

**Figure 15:**
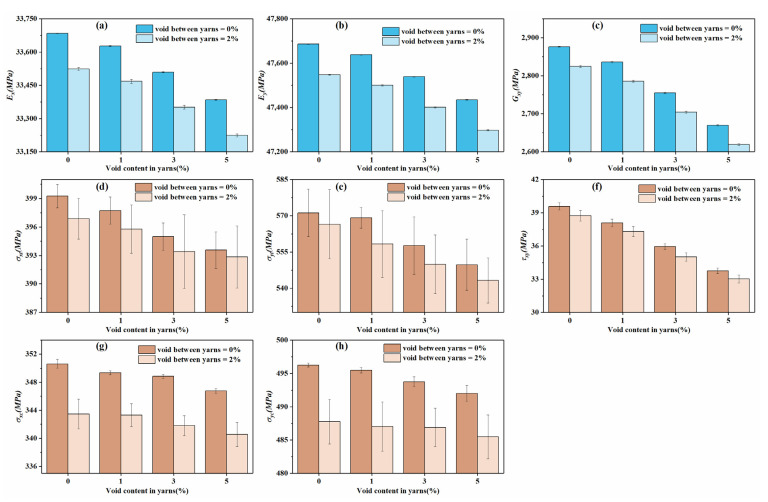
Statistical elastic properties and strength properties of mesoscale model in warp, weft and in-plane shear directions. (**a**–**c**) represent the elastic moduli of 3DOWCs; (**d**–**h**) represent the strength properties of 3DOWCs.

Figure 14 depicts the typical stress–strain curves of 3DOWCs with no voids under different loading directions. From the image, the initial slopes of tensile and compressive stress–strain curves were consistent; therefore, the elastic moduli were obtained from the tensile stress–strain curves. Figure 15 presents the statistical results for elastic properties and strength properties in the warp, weft and in-plane shear directions. The void contents in fiber bundles were set as 0%, 1%, 3% and 5%, which are the same as those of the microscale models. Void contents of 0% and 2% were considered between bundles. The fiber volume content remained constant in all models. From the figures, the modulus and strength properties all exhibited a decreasing trend as the void content increased. Figure 15a–c illustrates the relationships between the modulus and void content. Even though the presence of voids reduced the moduli in the warp, weft and in-plane shear directions, the decreasing amplitude was unnoticeable. Moreover, the variance of the moduli was not significant. Taking the void-free case as an example, the minimum and maximum moduli in the warp direction were 33,679.79 MPa and 33,687.56 MPa, respectively. In the microscale model, the standard deviation of the longitudinal modulus was small, while that of the transverse modulus was relatively large. The void had nearly no influence on the longitudinal modulus, while the transverse modulus reduced by 9.35% when the void content was increased to 5%. In the mesoscale model, the 3DOWCs had longitudinal fiber tows in both the warp and weft directions, and the longitudinal fiber tows were the main load-bearing components. Different from the results for modulus, the models with randomly distributed element properties showed significant variation in strength. With the uneven materials distributed in fiber bundles, some elements had high tensile or compressive strengths, while others had lower ones. Low-strength properties resulted in the early failure of the elements. Evaluation using the Puck’s criterion revealed that the elements exhibited reduced stiffness, lost the load-bearing capacity and featured stress concentration, which may further propagate the damage process. However, the elements with low strength properties may not be located at the key positions or in the load directions; if so, the overall strengths of the 3DOWCs will be relatively high. Moreover, the variations in mechanical properties, including moduli and strengths, were generally remarkable when voids were randomly distributed in the matrix between bundles because of the inclusion of one more uncertainty source.

**Figure 16:**
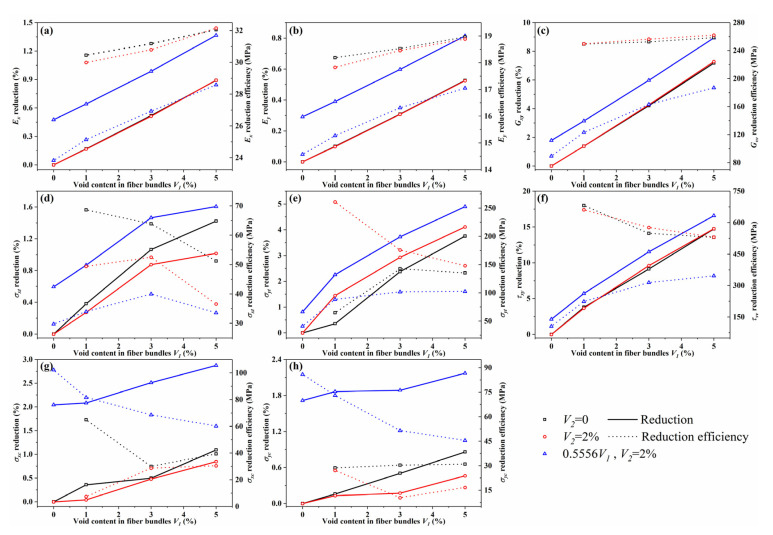
Reduction and reduction efficiency of mechanical properties for mesoscale models. (**a**–**c**) represent the modulus reduction and reduction efficiency; (**d**–**h**) represent the strength reduction and reduction efficiency.

From Figure 15, the randomly distributed voids in fiber bundles and between bundles caused different degrees of reduction in modulus and strength. To further evaluate the influence of voids on these mechanical properties, the reductions were calculated using the mean values. The total content of fiber bundles in the mesoscale model was 55.56%; thus, the void content in the microscale model should be translated to the mesoscale model by multiplying the void content by 0.5556. Assume $V_{1}$ is the void content in fiber bundle, then $0.5556V_{1}$ denotes the void content in the fiber bundles relative to the whole 3DOWC model. $V_{2}$ means the void content between fiber bundles and it is relative to the whole composite. For the convenience of subsequent descriptions, solid, dashed, black, red and blue lines or symbols are used to describe the various cases. The solid lines and dashed lines denote the reduction and reduction efficiency of the corresponding mechanical parameter at different void content cases. Black symbols describe the $V_{2}=0$ cases, and the baseline is $\left(V_{1},V_{2}\right)=(0,0)$. It denotes there is no void between fiber bundles and only voids inside the bundles are considered. Red symbols describe the $V_{2}=2\%$ cases, and the baseline is $\left(V_{1},V_{2}\right)=(0,2\%)$. Blue symbols denote the $V_{2}=2\%$ cases, but the baseline is $\left(V_{1},V_{2}\right)=(0,0)$. The difference between red and blue symbols is that they have different baselines. In Figure 16a–c, the square and circle solid lines almost coincide, which demonstrates that the voids between bundles did not influence the effect of voids in bundles on the elastic properties; that is, the modulus decreased to the same extent as the increase in the void content inside the fiber bundle for different $V_{2}$ cases. The decrease rate increased linearly with the increase in the void content inside the fiber bundles. In Figure 16, the blue solid line is almost parallel to the red and black solid line; this indicates that the reduction in modulus caused by the increase in the void content between fiber bundles was the same when the void content inside the fiber bundle was constant. The reduction efficiency is the corresponding mechanical parameter reduced per unit void content compared with the baseline. The void content here represents the total voids in 3DOWC including voids inside and between fiber bundles. Even though the pores between and inside the fiber bundles exhibited a linear reduction in the modulus, the efficiencies of the modulus reduction for both void contents were not the same. As illustrated in the figure, the black and red dashed lines do not differ much for a certain $V_{2}$, but the blue dashed line is significantly smaller than the black and red dashed lines, indicating that the effect of voids between fiber bundles on the 3DOWC modulus was smaller than the effect of pores inside fiber bundles with the same content on the 3DOWC modulus. Moreover, the blue dashed line tends to rise more slowly. The effect of the two types of voids on the strength properties was much more complicated than that on the modulus properties. For the tensile strength, $\sigma_{xt}$ increased with $V_{1}$ at a certain $V_{2}$, but the increase tended to decrease after 3% void content. As seen from the dashed line, the black line had the highest reduction efficiency, and the reduction in strength due to voids became less significant with the appearance of microscale void defects, indicating that the voids inside the fiber bundle had a greater effect on the 3DOWC tensile strength, and the internal defects were more likely to cause a reduction in the 3DOWC strength. However, for $\sigma_{xc}$, the blue curve has the highest reduction efficiency, which indicates that the voids between fiber bundles had a greater effect on the overall compressive strength under compression in the warp direction. Similar conclusions can be reached for $\sigma_{yc}$. According to [48,49], the tensile strength of 3D woven composites is strongly influenced by the fiber bundle properties, while the compressive properties are largely controlled by the matrix properties. In this study, the internal voids of fiber bundles caused the reduction of the properties of 3DOWC fiber bundle components, while the mesoscale voids caused the reduction of the properties of 3DOWC matrix components. These findings are consistent with the results in the literature [48,49] that intra-fiber bundle voids have a greater effect on tensile and shear properties, while the mesoscale defects have a greater effect on compressive strength properties. In the current study, for shear strength properties, the decrease in $\tau_{s12}$ with increasing $V_{1}$ was the same for the same $V_{2}$ and was approximately linear in the given range of void contents. At the same $V_{2}$, the $\tau_{s12}$ reduction efficiency tended to decrease with increasing $V_{1}$ and eventually tended to level off. At $V_{2}=2\%$, the overall $\tau_{s12}$ reduction efficiency was significantly lower than that at $V_{2}=0$, but the reduction efficiency featured a growing trend and eventually leveled off, which is inconsistent with the trend of the black line, indicating that coupling effects that affect the shear strength performance existed between the two kinds of voids.

In general, the void content inside the fiber bundle and between the fiber bundles both exhibited a roughly linear decreasing trend on the modulus, and the effect of the voids inside fiber bundles was higher than that of the voids between the fiber bundles. The two defects seemed to be independent of each other, with no significant coupling effect. Regarding strength properties, coupling relationships existed between the effects caused by the two defects, and the presence of voids between fiber bundles changed the pattern of the effect of voids inside fiber bundles on strength properties. Moreover, the internal voids had a greater effect on tensile and shear properties, but the compressive strength was more influenced by the mesoscale voids.

## 5. Conclusions

In this paper, a multiscale analysis is proposed to evaluate the influence of micro- and meso-void defects on the elastic properties and strength properties of 3DOWCs. Randomly distributed fiber filaments and voids were considered in the microscale model. The outputs from lower-scale models were utilized as inputs for higher-scale models. Non-uniform material properties were assigned to the fiber bundle elements of 3DOWCs. Statistical results are presented to provide an insight into the effect of voids on the mechanical performance of the composites.

In the microscale analysis, 150 randomly distributed fiber filaments models were established, and three models with randomly distributed voids were generated for each void-free model and each void content. Mean values and standard deviations were calculated to provide intuitive results of mechanical properties. The relationships between the two properties were obtained by plotting the bubble figures between any two parameters. The mean values and dispersity of the material properties could be considered to describe the relationships through linear fitting and the generation of random errors. The proposed method ensures the reasonableness of newly generated material properties. The generated parameters agree well with the original calculated results.

In the microscale analysis, a voxel finite element model for 3DOWC was established. Generated material properties using the previous method were assigned for the fiber bundle properties as uncertainty sources from microscale models. Another uncertainty source was the void defects between bundles. Extended Puck’s criterion was implemented to predict the failure initiation, and an energy-based damage evolution model was adopted for the progressive damage processes. The elastic properties and strength properties in the warp/weft tension/compression and in-plane shear directions were calculated. The results indicate the following: (1) the void defects will reduce the elastic properties, but the variations are not sensitive to the uncertainties; (2) the strength properties are sensitive to the uncertainties caused by the non-uniform distributed material properties of fiber bundles; (3) the elastic properties roughly linearly decreased with the void contents, including voids in and between fiber bundles; (4) coupling effects that affect the strength properties occurred between the two kinds of void defects; and (5) tensile and shear strengths were sensitive to the voids inside the bundles, while the compressive strength was sensitive to the voids between bundles.

The present research can provide the design basis for evaluating the influence of two kinds of void defects on 3DOWC mechanical properties. In future work, a similar procedure will be conducted for macroscale models, such as 3D woven structures, considering not only the present uncertainties but also the bundle section uncertainties.

## Figures and Tables

**Table 1:** Mechanical properties of fiber and matrix.

	$E_{1}/\mathrm{GPa}$	$E_{2}/\mathrm{GPa}$	$\upsilon_{12}$	$G_{12}/\mathrm{GPa}$	$G_{23}/\mathrm{GPa}$	$\sigma_{xt}/\mathrm{MPa}$	$\sigma_{xc}/\mathrm{MPa}$
Fiber	231	12.97	0.3	11.28	4.45	3350	2500
Matrix	3.5		0.35			103.4	241

## Data Availability

Data is contained within the article.

## Appendix A

**Table A1:** Mean value and standard deviation of all mechanical properties for micro-scale models.

	Void	Mean	Std		Void	Mean	Std		Void	Mean	Std
$E_{1}$ (MPa)	0	138,553.96	5.304	$\sigma_{xt}$ (MPa)	0	2022.16	0.251	$G_{IC}^{xt}$ (N/mm)	0	15.16	5.8 × 10^−4^
	1%	138,513.33	5.4		1%	2005.18	2.664		1%	15.1	0.0236
	3%	138,433.2	5.583		3%	2002.2	0.158		3%	14.99	0.02594
	5%	138,353.58	5.736		5%	2001.07	0.243		5%	14.9	0.02473
$E_{2}$ (MPa)	0	7558.85	92.336	$\sigma_{xc}$ (MPa)	0	1503.3	0.18	$G_{IC}^{xc}$ (N/mm)	0	8.256	2.81 × 10^−4^
	1%	7423.79	95.367		1%	1502.14	0.365		1%	8.245	0.00364
	3%	7149.63	96.21		3%	1500.29	1.807		3%	8.238	0.00876
	5%	6852.07	109.8		5%	1497.43	2.274		5%	8.235	0.01295
$\upsilon_{12}$	0	0.318	4.038 × 10^−4^	$\sigma_{yt}$ (MPa)	0	64.07	2.053	$G_{IC}^{yt}$ (N/mm)	0	0.426	0.0358
	1%	0.3172	4.007 × 10^−4^		1%	57.41	4.512		1%	0.394	0.0352
	3%	0.3156	4.019 × 10^−4^		3%	49.17	4.142		3%	0.346	0.0344
	5%	0.3139	4.454 × 10^−4^		5%	43.69	3.505		5%	0.315	0.032
$\upsilon_{23}$	0	0.4755	0.00602	$\sigma_{yc}$ (MPa)	0	147.2	5.299	$G_{IC}^{yc}$ (N/mm)	0	2.236	0.1241
	1%	0.4728	0.00619		1%	131.78	11.065		1%	1.809	0.1482
	3%	0.4664	0.00709		3%	112.3	10.002		3%	1.777	0.141
	5%	0.4597	0.00871		5%	99.53	8.314		5%	1.585	0.1409
$G_{12}$ (MPa)	0	3876.98	73.309	$\tau_{12s}$ (MPa)	0	50.91	2.637	$G_{IC}^{12}$ (N/mm)	0	0.581	0.0548
	1%	3790.19	76.972		1%	48.92	2.695		1%	0.554	0.0549
	3%	3621.21	75.79		3%	45.54	2.705		3%	0.52	0.0493
	5%	3445.74	77.938		5%	41.9	2.389		5%	0.479	0.0475
$G_{23}$ (MPa)	0	2528.02	38.013	$\tau_{23s}$ (MPa)	0	60.06	4.352	$G_{IC}^{23}$ (N/mm)	0	1.381	0.1438
	1%	2486.08	38.284		1%	55.17	4.032		1%	1.253	0.1214
	3%	2396.78	40.006		3%	50.35	3.117		3%	1.135	0.099
	5%	2306.22	47.622		5%	46.08	3.126		5%	1.026	0.0956

## References

[1] Fuchs E.R.H., Field F.R., Roth R., Kirchain R. (2008). Strategic materials selection in the automobile body: Economic opportunities for polymer composite design. Compos. Sci. Technol..

[2] Huang T., Wang Y., Wang G. (2018). Review of the Mechanical Properties of a 3D Woven Composite and Its Applications. Polym. -Plast. Technol. Eng..

[3] Su Z., Huang F., Liu Q. (2021). Development of low cost out-of-autoclave Molding Technology for Advanced Composite Materials. Hi-Tech. Fiber Appl..

[4] Almeida S., Neto Z.D.S.N. (1994). Effect of void content on the strength of composite laminates. Compos. Struct..

[5] Zhang A., Lu H., Zhang D. (2016). Research on the mechanical properties prediction of carbon/epoxy composite laminates with different void contents. Polym. Compos..

[6] Di Landro L., Montalto A., Bettini P., Guerra S., Montagnoli F., Rigamonti M. (2017). Detection of Voids in Carbon/Epoxy Laminates and Their Influence on Mechanical Properties. Polym. Polym. Compos..

[7] Tao W., Zhu P., Xu C., Liu Z. (2020). Uncertainty quantification of mechanical properties for three-dimensional orthogonal woven composites. Part II: Multiscale simulation. Compos. Struct..

[8] Šejnoha M., Zeman J. (2006). Micromechanical modeling of random or imperfect composites. WIT Trans. Built Environ..

[9] Šejnoha M., Zeman J. (2008). Micromechanical modeling of imperfect textile composites. Int. J. Eng. Sci..

[10] Puck A., Kopp J., Knops M. (2002). Guidelines for the determination of the parameters in Puck’s action plane strength criterion. Compos. Sci. Technol..

[11] Potter K., Khan B., Wisnom M., Bell T., Stevens J. (2008). Variability, fibre waviness and misalignment in the determination of the properties of composite materials and structures. Compos. Part A Appl. Sci. Manuf..

[12] Mesogitis T.S., Skordos A.A., Long A.C. (2014). Uncertainty in the manufacturing of fibrous thermosetting composites: A review. Compos. Part A Appl. Sci. Manuf..

[13] Gao X., Yuan L., Fu Y., Yao X., Yang H. (2020). Prediction of mechanical properties on 3D braided composites with void defects. Compos. Part B Eng..

[14] Shi D., Teng X., Jing X., Lyu S., Yang X. (2020). A multi-scale stochastic model for damage analysis and performance dispersion study of a 2.5D fiber-reinforced ceramic matrix composites. Compos. Struct..

[15] Wang X.F., Wang X.W., Zhou G., Zhou C. (2007). Multi-scale Analyses of 3D Woven Composite Based On Periodicity Boundary Conditions. J. Compos. Mater..

[16] Zhou Y., Lu Z., Yang Z. (2013). Progressive damage analysis and strength prediction of 2D plain weave composites. Compos. Part B Eng..

[17] Vajari D.A., González C., Llorca J., Legarth B.N. (2014). A numerical study of the influence of microvoids in the transverse mechanical response of unidirectional composites. Compos. Sci. Technol..

[18] Vajari D.A. (2015). A micromechanical study of porous composites under longitudinal shear and transverse normal loading. Compos. Struct..

[19] Wang M., Zhang P., Fei Q., Guo F. (2019). Computational evaluation of the effects of void on the transverse tensile strengths of unidirectional composites considering thermal residual stress. Compos. Struct..

[20] Paiboon J., Griffiths D., Huang J., Fenton G.A. (2013). Numerical analysis of effective elastic properties of geomaterials containing voids using 3D random fields and finite elements. Int. J. Solids Struct..

[21] Griffiths D.V., Paiboon J., Huang J., Fenton G.A. (2012). Homogenization of geomaterials containing voids by random fields and finite elements. Int. J. Solids Struct..

[22] Suo Y., Wang B., Jia P., Gong Y. (2020). The effect of fabrication defects on the mechanical behaviors of metal matrix composites. Mater. Today Commun..

[23] Tao W., Zhu P., Xu C., Liu Z. (2020). Uncertainty quantification of mechanical properties for three-dimensional orthogonal woven composites. Part I: Stochastic reinforcement geometry reconstruction. Compos. Struct..

[24] Zhou L.C., Chen M.W., Liu C., Wu H.A. (2018). A multi-scale stochastic fracture model for characterizing the tensile behavior of 2D woven composites. Compos. Struct..

[25] Rafiee R., Torabi M.A. (2018). Stochastic prediction of burst pressure in composite pressure vessels. Compos. Struct..

[26] Guo J., Wen W., Zhang H., Cui H., Song J. (2020). Representative cell modeling strategy of 2.5D woven composites considering the randomness of weft cross-section for mechanical properties prediction. Eng. Fract. Mech..

[27] Dong C. (2016). Effects of Process-Induced Voids on the Properties of Fibre Reinforced Composites. J. Mater. Sci. Technol..

[28] Jiang H., Ren Y., Liu Z., Zhang S. (2018). Microscale finite element analysis for predicting effects of air voids on mechanical properties of single fiber bundle in composites. J. Mater. Sci..

[29] Carrera E., Petrolo M., Nagaraj M., Delicata M. (2020). Evaluation of the influence of voids on 3D representative volume elements of fiber-reinforced polymer composites using CUF micromechanics. Compos. Struct..

[30] Hyde A., He J., Cui X., Lua J., Liu L. (2020). Effects of microvoids on strength of unidirectional fiber-reinforced composite materials. Compos. Part B Eng..

[31] Dong J., Huo N. (2016). A two-scale method for predicting the mechanical properties of 3D braided composites with internal defects. Compos. Struct..

[32] Huang T., Gong Y. (2018). A multiscale analysis for predicting the elastic properties of 3D woven composites containing void defects. Compos. Struct..

[33] Lin H., Brown L.P., Long A.C. (2011). Modelling and Simulating Textile Structures Using TexGen. Adv. Mater. Res..

[34] Liu Y., Straumit I., Vasiukov D., Lomov S.V., Panier S. (2017). Prediction of linear and non-linear behavior of 3D woven composite using mesoscopic voxel models reconstructed from X-ray micro-tomography. Compos. Struct..

[35] Barbero E.J. (2013). Finite Element Analysis of Composite Materials Using Ansys^®^.

[36] Li S., Warrior N., Zou Z., Almaskari F. (2011). A unit cell for FE analysis of materials with the microstructure of a staggered pattern. Compos. Part A Appl. Sci. Manuf..

[37] Lubliner J., Oliver J., Oller S., Onate E. (1989). A plastic-damage model for concrete. Int. J. Solids Struct..

[38] Lee J., Fenves G.L. (1998). Plastic-Damage Model for Cyclic Loading of Concrete Structures. J. Eng. Mech..

[39] Puck A., Schürmann H. (1998). Failure analysis of frp laminates by means of physically based phenomenological models. Compos. Sci. Technol..

[40] Gu J., Chen P. (2018). Extension of Puck’s inter fibre fracture (IFF) criteria for UD composites. Compos. Sci. Technol..

[41] Deuschle H.M., Puck A. (2013). Application of the Puck failure theory for fibre-reinforced composites under three-dimensional stress: Comparison with experimental results. J. Compos. Mater..

[42] Knops M. (2008). Analysis of Failure in Fiber Polymer Laminates: The Theory of Alfred Puck.

[43] Deuschle H.M., Kröplin B.-H. (2012). Finite element implementation of Puck’s failure theory for fibre-reinforced composites under three-dimensional stress. J. Compos. Mater..

[44] Schirmaier F.J., Weiland J., Kärger L., Henning F. (2014). A new efficient and reliable algorithm to determine the fracture angle for Puck’s 3D matrix failure criterion for UD composites. Compos. Sci. Technol..

[45] Thomson D.M., Cui H., Erice B., Hoffmann J., Wiegand J., Petrinic N. (2017). Experimental and numerical study of strain-rate effects on the IFF fracture angle using a new efficient implementation of Puck’s criterion. Compos. Struct..

[46] Tuo H., Lu Z., Ma X., Zhang C., Chen S. (2019). An experimental and numerical investigation on low-velocity impact damage and compression-after-impact behavior of composite laminates. Compos. Part B Eng..

[47] Duvaut G., Lions J.L., John C.W., Cowin S.C. (1977). Inequalities in Mechanics and Physics. J. Appl. Mech..

[48] Cox B.N., Dadkhah M.S., Inman R.V., Morris W.L., Zupon J. (1992). Mechanisms of compressive failure in 3D composites. Acta Metall. Mater..

[49] Cox B.N., Dadkhah M.S., Morris W.K., Flintoff J.G. (1994). Failure mechanisms of 3D woven composites in tension, compression, and bending. Acta Metall. Materialia..

